# Gendermetrics of cancer research: results from a global analysis on prostate cancer

**DOI:** 10.18632/oncotarget.24716

**Published:** 2018-04-13

**Authors:** Michael H.K. Bendels, Alecsandru M. Costrut, Norman Schöffel, Dörthe Brüggmann, David A. Groneberg

**Affiliations:** ^1^ Division of Computational Medicine, The Institute of Occupational, Social and Environmental Medicine, Goethe University, Frankfurt, Germany; ^2^ Department of Obstetrics and Gynecology, Keck School of Medicine of University of Southern California, Los Angeles, CA, USA

**Keywords:** sex, bibliometry, authorship, citation, productivity

## Abstract

**Background:**

The present study aims to elucidate the success concerning gender equality in cancer research in the last decade (from 2008 to 2017) with prostate cancer as the target parameter.

**Results:**

31.7% of all authorships and 36.3% of the first, 32.5% of the co- and 22.6% of the last authorships were held by women. The corresponding female-to-male odds ratio is 1.26 (CI: 1.22–1.30) for first, 1.15 (CI: 1.12–1.18) for co- and 0.59 (CI: 0.57–0.62) for last authorships. The annual growth rates are 0.6% overall and 0.9% for first, 0.2% for co-authorships, and 2.8% for last authorships. Women are slightly underrepresented at prestigious authorships compared to men. The female underrepresentation accentuates in articles with many authors that attract the highest citation rates. Multi-author articles with male key authors are more frequently cited. Men publish more articles compared to women (61.8% male authors are responsible for 68.3% of the authorships) and are overrepresented at productivity levels of more than 1 article per author. Major regional differences were found with best female odds in Sweden, Brazil, and Austria. The prognosis for the next decade forecasts a harmonization of authorship odds.

**Conclusion:**

Prostate cancer research is characterized by a career dichotomy with few women in academic leadership positions and many female early career researchers. This career dichotomy has been narrowed in the last decade and will likely be further reduced in the future.

**Methods:**

On the basis of the Gendermetrics Platform, a total of 26,234 articles related to prostate cancer research were analyzed.

## INTRODUCTION

Prostate cancer is the most common male cancer from a global viewpoint. It is also the second leading cause of cancer death among men in the USA [1]. According to the GLOBOCAN (2012) project of the International Association of Cancer Registries (IACR), an estimated 1.1 million new cases and 307,000 deaths were reported in 2012 [1]. With this enormous global burden of diseases, prostate cancer is in the focus of many research initiatives ranging from epidemiological and genetic assessments [2–8] over diagnostic issues [9–14] to therapy [15–19] and even prevention [20]. But there are numerous more implications: For instance, there is a general consensus about a major gender imbalance in academic urology, the field of medicine that drives the motor of clinical and translational research for prostate cancer. Han *et al*. recently summarized [21] that women are disproportionately underrepresented when it comes administrative leadership positions in urology. However, they also state that the gender gap is narrowing since more women are pursuing careers in urology [21]. They conclude that there may be more women in positions of leadership over time [21]. Within the field of urology, prostate cancer belongs to the most important oncological entities and it is enticing to speculate if the gender distribution in this area of research follows the findings of Han *et al*. Thus, we here conducted a bibliometric study that analysis the gender distribution in prostate cancer research.

Methodically, we assessed the integration of women by analyzing their representation in scientific authorships. In medicine, the prestige of authorships follows, by convention, a ranked order with a higher reputation of first and last authorships and a lower reputation of co-authorships [22–24]. Moreover, hierarchical structures of the research groups are reflected by authorships, as early-career researches usually publish as first or co-authors, while senior researches prefer the last author position [22, 24, 25].

Methodically, we applied the Gendermetrics Platform [26] to analyze the representation of 148,721 male and female authorships from 19,724 English original articles related to prostate cancer that were published between January 1, 2008 and September 12, 2017. By including the different prestige of first, co- and last authorships, we draw conclusions about the distribution of prestigious authorships between the two genders, as previously shown in Bendels *et al*. [23, 24, 27]. The analysis evaluates global status, temporal development and future perspectives, gender-specific differences across countries, in scholarly productivity, citation rates and finally, the role played by women in respect of articles with multiple authors, e.g. collaborative articles [24].

## RESULTS

### Female authorships on the global level

**Figure 1:**
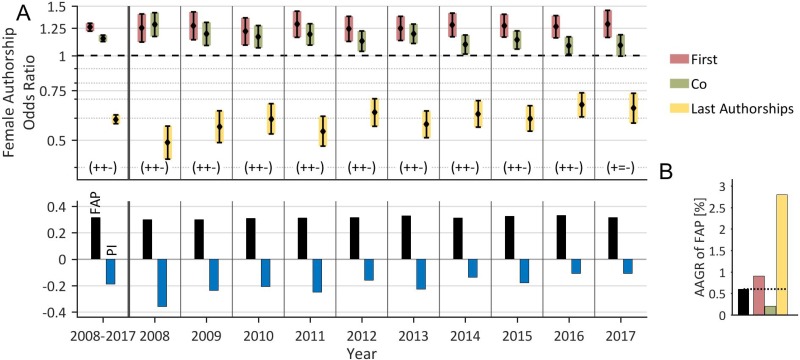
Time trend of female authorships on the global level (**A**) The proportion of female authorships (FAP, bottom), the pattern of female authorship odds (FAOR with FAOR-tuple, top) and the associated *Prestige Index* (PI) are depicted by year and averaged over time. The FAOR-patterns are predominantly characterized by significant (*P* < .05) higher female odds for first- or co-authorships and lower odds for last authorships compared to men (FAOR-tuple (+, +, –)). The FAP is on average 31.7%. The *Prestige Index* is on average negative (-0.19), but exhibits a distinct upward trend during the last decade. (**B**) The FAP shows a small increase during the period as indicated by its annual growth rate (AAGR) of 0.6%. The highest AAGR was revealed for last authorships (2.8%).

As a first step, evolution over time of female authorships in the field of prostate cancer on a global level was analyzed (Figure 1). We showed that female authorships were underrepresented, with a total FAP of 31.7%. Of these, there were relatively more female first (36.3%) authorships and co-authorships (32.5%) but a substantially lower proportion of last authorships (22.6%). The FAP grew from 29.9% in 2008 to 31.6% in 2017; giving an AAGR of 0.6% (Figure 1B). The highest AAGR was for last authorships (2.8%), followed by first authorships (0.9%) and co-authorships (0.2%).

The global pattern of FAORs is illustrated by the FAOR-triplet (+, +, –), i.e. there are significantly higher odds ratios for females to secure first authorships, (1.26, CI: 1.22–1.30) and co-authorships (1.15, CI: 1.12–1.18), but there are significantly lower odds ratios for females to secure last authorships (0.59, CI: 0.57–0.62). The FAOR-triplet has been almost constant over the whole evaluation period (Figure 1A). The imbalance of odds between genders for authorships is reflected in the *Prestige Index*, the average of which is 0.19, which indicates that females have slightly worse odds of securing prestigious authorships compared to men. The *Prestige Index* shows an increase from –0.36 in 2008 to –0.11 in 2017 which represents a substantial improvement in the odds for females securing prestigious authorships (Figure 1A, bottom).

### Differences across countries

When we refined our analysis from the global level to a country-specific level, we identified a wide range of FAPs in prostate cancer research which ranged from 11.1% in Japan to 50.0% in Spain (Table 1). The most unfavorable FAOR-triplet (=, +, –) was found in Greece, France, and Italy, whereas Sweden, Brazil, and Austria are all characterized by the more favorable FAOR-triplet (+, –, =). Norway provides an example of gender-neutrality in respect to all types of authorship (FAOR-triplet (=, =, =)). The highest *Prestige Indices* were calculated for Sweden (0.96), Brazil (0.38), and Austria (0.35). The lowest *Prestige Indices* were found in Greece (–1.47), Japan (–0.86) and France (–0.39). We found no significant correlation between a country's FAP and its *Prestige Index* (r(19)=.10, *P* > .05).

**Table 1:** Classification of countries (descendingly ordered by the *Prestige Index*)

Country Name	Prestige Index	FAP	FAOR Triplet	No. Articles	No. Authorships
Sweden	0.96	29.7%	(+, –, =)	919	2,843
Brazil	0.38	41.4%	(+, –, =)	300	1,185
Austria	0.35	23.0%	(+, –, =)	319	1,320
Switzerland	0.28	25.9%	(+, =, =)	483	1,554
Poland	0.24	43.9%	(+, =, –)	241	997
Netherlands	0.23	31.5%	(+, –, –)	930	3,215
Belgium	0.1	28.0%	(+, =, =)	385	1,215
Norway	0.09	42.2%	(=, =, =)	308	1,189
Australia	0.02	40.1%	(=, =, =)	993	4,255
Finland	–0.04	37.7%	(+, =, –)	482	1,799
Denmark	–0.04	38.9%	(+, =, –)	401	1,364
United Kingdom	–0.18	37.6%	(+, =, –)	1,989	8,309
Spain	–0.18	50.0%	(=, =, –)	745	2,894
Turkey	–0.19	27.6%	(=, =, –)	343	1,313
Canada	–0.2	30.3%	(+, +, –)	1,936	7,606
Germany	–0.26	26.1%	(+, +, –)	2,148	10,256
United States	–0.36	31.7%	(+, +, –)	11,541	58,510
Italy	–0.37	40.9%	(=, +, –)	1,585	9,268
France	–0.39	31.5%	(=, +, –)	1,319	4,350
Japan	–0.86	11.1%	(+, +, –)	1,578	9,943
Greece	–1.47	37.0%	(=, +, –)	243	779

### Female authorships by the number of authors per article

**Figure 2:**
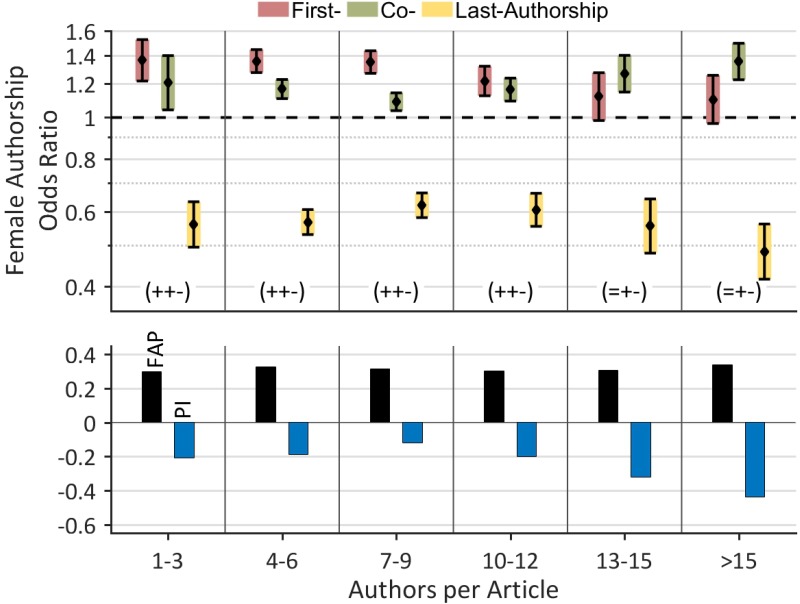
Female authorships by authors per article The female odds to secure prestigious authorships increase for a small number of authors per article. This trend is reversed for articles with more than 9 authors. The lowest Prestige Index (–0.44) was revealed for articles with more than 15 authors. Thus, the female odds to secure prestigious authorships get worse in articles with many authors (e.g. collaboration projects). The FAP increases from 29.9% for articles with 1–3 authors to 34.0% for articles with more than 15 authors.

The FAP/FAOR-classification was also used to investigate the role normally played by women in respect of articles with multiple authors, e.g. collaborative articles (Figure 2) [24]. Firstly, the FAP increases from 29.9%, for articles with between 1 and 3 authors, and to 34.0% for articles with more than 15 authors. Secondly, we found that where there is a high number of authors (more than nine authors per article), the FAORs for prestigious first or last authorships shows a continual decrease (first: from 1.35 for 7–9 authors to 1.10 for >15 authors, last: from 0.62 to 0.48), whereas the FAOR for, less prestigious, co-authorships increases (from 1.09 to 1.36). Overall, this leads to a continual decrease in the *Prestige Index* from –0.12 for articles with 7–9 authors, to –0.64 for articles with more than 15 authors. In conclusion, it appears that the odds for females securing prestigious authorships are worse for articles with many authors (e.g. collaborative projects).

### Citation and productivity analysis

**Figure 3:**
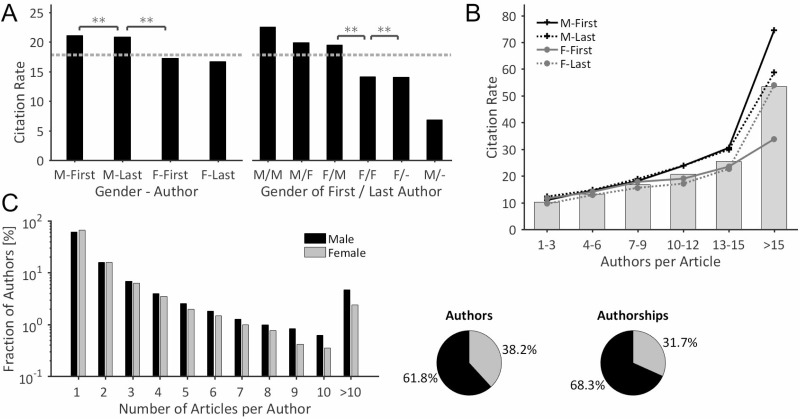
Gender-specificity of citations & scholarly productivity (**A**) The descendingly ordered citation rates document that male-authored articles are more frequently cited than female-authored articles. The dotted line characterizes the mean citation rate of 17.8 citations/article (Kruskal-Wallis test, ^*^P < .05 ^**^P < .01). (**B**) Average citation rates of both, ungrouped articles (bars) and articles that were grouped by the gender of their key authorships (lines), plotted with respect to the number of authors. The citation rate of an article is higher the more authors are involved. Significant differences in citation rates between the two genders emerge for articles with more than nine authors per article. (**C**) Gender-specific distribution of the number of articles per author. Women dominate the sub-group ‘author has 1 article’. All other sub-groups show a relative over-representation of male authors, which accentuates with increasing productivity levels. Overall, female authors have a lower productivity, as 38.2% female authors are responsible for 31.7% of all authorships.

When the citation rates are considered in a gender-specific way, articles with male first- and last-authors are cited most often with average citation rates of 21.1 citations per article for articles with a male first author, and 20.8 for those with a male last author (Figure 3A, left). By contrast, articles with female first authors have an average citation rate of 17.2 citations per article while those with a female last author average 16.7 citations per article; both these figures are below the mean citation rate of 17.8 citations per article. This means that articles with male key authors are more frequently cited than those with female key authors. There are statistically significant differences when the articles are grouped by gender regardless of the author's position.

When combined key authorships (first/last authorship) are analyzed, it can be seen that ‘male/male’ articles, with male first and last authors, have on average the highest citation rates with 22.5 citations per article, followed by articles with male first and female last authors (19.9 citations per article), female first and male last authors (19.5 citations per article), and female first and last authors (14.1 citations per article) (Figure 3A, right). On average, articles with a single female author are cited more frequently (14.1 citations per article) than articles with a single male author (6.8 citations per article). The differences are however not statistically significant. In this category, only multi-author articles with a male first or last author are above the mean citation rate.

Statistically, the citation rate of an article increases dependent on the number of authors involved (Figure 3B), for example, articles with 1 to 3 authors have an average citation rate of 10.2, while articles with more than 15 authors have an average citation rate of 53.4. There were no significant gender-specific differences in citation rates for articles with up to 9 authors. When articles have more than 9 authors, significant differences in citation rates between the two genders emerge, this is particularly apparent between articles with male last authors which are cited on average 74.6 times, and those with female first authors that are cited on average 33.8 times (Figure 3B).

Marked differences in scientific productivity between the two genders are shown by the analysis: Women clearly dominate the sub-group with the lowest productivity (author has one single article), as 66.1% of the female authors, but only 60.7% of the male authors in our dataset had only published one article (Figure 3C). For all other sub-groups, where authors had published more than one article, we found a clear over-representation of male authors, which rose with increasing productivity. In particular, the sub-group of ‘most productive authors’ in which authors had published more than 10 articles, is dominated by men, as 4.6% of the male authors but only 2.4% of the female authors had published more than 10 articles [23]. In total, 61.8% male authors are responsible for 68.3% of all authorships in our data set, thus indicating the male scholars’ higher productivity.

## DISCUSSION

### Career dichotomy

In this analytical study, a bibliometric approach was used to investigate the representation of women in prostate cancer research. The global FAP of 31.7%, is comparable to the estimate of 30% for science generally by Lariviere *et al*. in 2012 [28] and 31.3% for the field of lung cancer research [24]. The value is significantly lower compared to the FAPs of 34.0% shown for six high-impact medical journals [29] and the fields of dermatology (43.0%, unpublished data), epilepsy (39.4%) [23], schizophrenia (37.6%) [27], and stroke medicine (36.3%, unpublished data) research for the same period.

There is an uneven distribution of women across the different authorships: Compared to men, we found women to be relatively overrepresented in first and co-authorships and to be underrepresented in last authorships (FAOR-pattern (+, +, –)). This pattern appears to reflect the well-known male-female dichotomy in scientific careers, in which there are many female researchers at lower levels in the hierarchy early in their careers and there are only a few women in leadership positions [23, 24, 27, 30–34].

The FAOR-distribution also shows that women are slightly underrepresented in prestigious authorships compared to men. As in other research areas, for example, lung cancer, epilepsy or schizophrenia research [23, 24, 27], the high FAOR for first-authorships does not compensate the unfavorable FAORs for co- and last authorships [23]. As academic publishing of prestigious authorships is the key element for career advancement in science, this is a very important result [27, 35–37]. Reasons for the relative overrepresentation of female co-authorships, which have been discussed by West *et al*. [38], range from the high influx of female early-career researchers in recent decades, through females’ lack of success in negotiating for more prestigious authorships, to speculations suggesting that women make a smaller contribution to an article [23, 24].

### Position effects productivity and citation rate

In prostate cancer research 38.2% female authors are responsible for 31.7% of the authorships which shows that, as in many other disciplines [23, 27, 28, 30, 32, 33, 39], fewer articles are published by women compared to men [23]. This mismatch is in a comparable range with other medical disciplines such as lung cancer research, where 37.8% female authors hold 31.3% of the authorships [24]. When considering the productivity of single authors, we were able to reproduce the marked overrepresentation of male authors at higher productivity levels, which has been previously shown in the fields of lung cancer research [24], evolutionary biology and ecology [23], epilepsy [23] and schizophrenia research [27]. One very probable reason for the higher productivity of male authors, is the higher output of the primarily male, senior scientists [27, 34] who are often members of a more or less fruitful scientific network. Due to these *structural reasons* [35], the underrepresentation of female authors of prestigious authorships increases for articles with many authors (Figure 2), e.g. for those highly competitive, collaborative articles, which have the highest citations rates (Figure 3B) [40]. It is plausible to assume that a result of this competitive displacement is the slightly higher citation rates for articles with male key authors compared to articles with female key authors, this is shown particularly in multi-author articles with up to 9 authors, which do not exhibit significant differences in citation rates between the two genders (Figure 3B). In terms of methodology, the time delay in the occurrence of citations (“Cited Half-Life”) [41], means that the results are biased towards the early period of investigation (2008–2010) [24].

### Country-specific aspects

We found significant differences between countries for both the proportion and odds ratios for female authorships. If the odds of securing prestigious authorships are taken as an indicator for career advancement in science [23, 24], then Sweden, Brazil, and Austria provided the best conditions for women. By contrast, Greece, Japan and France offered the optimal conditions for men in prostate cancer research. As we found a moderate linear correlation between a country's Prestige Index and its *Score,* as defined by the Global Gender Report (r(19)=.45, *P* < .05; Supplementary Figure 9), our findings correlate quite well with the results of the Global Gender Gap Report 2016 [42]. This suggests that the major regional differences are mainly due to socio-cultural and socio-economic conditions and do not result from discipline-specific factors [24]. Notably, no significant correlation between a country's FAP and its *Prestige Index* was found. This finding is consistent with results from the fields of lung cancer [24], epilepsy [23] and schizophrenia [27] research. This means that countries with a high FAP may fail to provide favourable career opportunities for women or vice versa [23]. A good example of this is Italy which has a low *Prestige Index* of –0.34 but a high FAP of 40.9%.

### Methodical limitations

The concept extended frequency-based methods [43–46] by including the odds ratios for female authorships [29] and the different prestige factors for first, co- and last authorships. The fully automated bibliometric methods ensure a fast and reliable analysis with minimisation of inter-individual variability. However, as already mentioned by Bendels *et al*. [23, 24], the scope of this method is limited because it does not include information regarding the scholar's academic position (e.g. Associate Professor vs. Full Professor), their academic degree, age and employment status. This information can only be gained by questionnaires or the inspection of e.g. online profiles, as has been done by other studies [33, 34, 45]. Another drawback of the bibliometric approach was that Asian countries with a high proportion of unisex names, for example, China, South Korea and Taiwan had to be excluded from the *country-specific* analysis.

## CONCLUSION AND OUTLOOK

**Figure 4:**
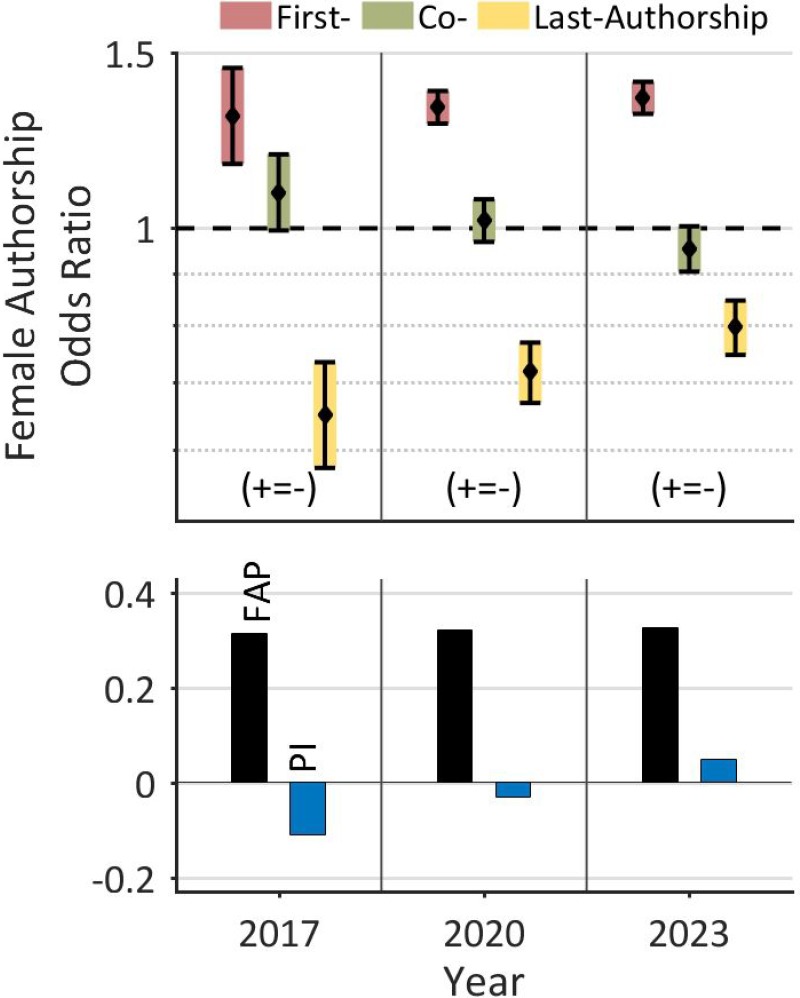
Linear projection of the development of female authorships on the global level The prognosis for the next years forecasts only a minor increase of the FAP (from 31.6% to 32.8%), but a harmonization of authorship odds between the two genders. According to this projection, the *Prestige Index* will become positive.

Overall, we found that, compared to other medical disciplines, there was a relatively low FAP [23, 27], a distinct male-female career dichotomy, and increased underrepresentation of females for key authorships of multi-author articles. On the other hand, the analysis also revealed a relatively high AAGR for female last authorships (2.8%). This finding reflects that women have been catching up during the last decade. A quantitative prognosis of the temporal development for female authorships up to the year 2023, based on this data, forecasts only a minor increase in the FAP (from 31.6% 2017 to 32.8% in 2023), but a significant improvement in the odds for female authorship (Figure 4). The detailed prognosis forecasts a harmonization of authorships odds between the two genders: an increase in the FAOR for first authorships (from 1.30 to 1.35) and last authorships (from 0.65 to 0.80) and a decrease in the FAOR for co-authorships (from 1.09 to 0.95). According to this projection, the *Prestige Index* will become positive (from -0.11 in 2017 to 0.05 in 2023). Therefore, and in line with a recent cross-sectional observational study of U.S. Urology Residency Programs in 2016–2017 [21], we do expect a deeper integration of women in the field of prostate cancer research with an increasing number of female leaderships in the next decade.

## METHODS

### Data acquisition and integration

We used the Gendermetrics platform [26] to assess the representation of women in prostate cancer research in a reliable and standardized way, see Bendels *et al*. [23, 24, 27]. For data collection, the Web of Science Core Collection (Thomson Reuters) was employed. The following title search term was generated: (TI = “Prostate cancer^*^” or TI = “Prostatic Neoplasm^*^” or TI = “Prostatic Cancer^*^”). The MeSH library (Medical Subject Headings) of the National Library of Medicine was used to generate the synonyms for ‘Prostate cancer’. The aim was to create a representative subset of prostate cancer related articles [23, 24, 27]. Following our protocol, we limited our search to English-language original research articles. The time frame was restricted from January 1, 2008 to September 12, 2017. In total, 26,234 articles were acquired. During data integration, authors were unified by names and first names yielding 85,378 authors.

### Gender determination

The Gendermetrics database was used to identify the authors’ genders [26]. In total, 34,421 (=40.3%) male authors, 21,312 (=25.0%) female authors, 9,275 (=10.9%) unisex authors and 20,370 (=23.9%) undefined authors were determined. The gender detection was numerically stable (Supplementary Figure 6) with a relatively little inter-annual variability (Supplementary Figure 2) and generates no bias towards a higher detection ratio of male or female names in our data set. Unisex and undefined authors and their authorships (*N* = 59,029) were ignored in further analysis [24]. The remaining *N* = 148,721 male and female authorships provide the data basis for the analysis. Following our protocol [23, 24], the research output of a country was assessed by considering the authorships of the associated institutions. According to the reality, a single author is thus able to contribute with various authorships to the research output of different countries (Supplementary Figure 8). In general, the quality of gender detection depends critically on the authorships’ country, as illustrated by Supplementary Figure 3 [23, 24]. In order to ensure the validity of the country-specific analysis, only countries with a detection fraction above 60.0% male and female authorships were included into this subanalysis. This threshold was chosen arbitrarily based on previous studies. Among the top 20 most productive countries, the Asian countries China, South Korea and Taiwan (with a high rate of unisex names) were excluded. Please note that the threshold criterion was exclusively applied for the country-specific analysis [23, 24]. Supplementary Figure 1 gives a general overview of the bibliometric data. Supplementary Figure 7 summarizes the methodical steps.

### Proportion of female authorships (FAP) & female authorship odds ratio (FAOR)

According to our study protocol [23, 24], three types of authorships were considered: First, co- and last authorships, whereby the term co-authorships encompasses all authorships between *one* first- and *one* last-authorship [23, 24]. Due to a lack of information, equally distributed first and last authorships were not considered. The definition of the proportion of female authorships (*FAP*) is “the quotient between the female authorship count and the total sum of male and female authorships” [23]. The author-specific odds ratios for female authors compared to male authors, are additionally determined (female authorship odds ratio, FAOR), with the corresponding confidence intervals at a confidence level of 95% [23]. The FAOR for first authorships is determined by considering all articles, however the FAORs for last and co-authorships are calculated differently by considering all articles from all authors credited with at least two or three authorships. A triplet was introduced as a system of indicating, by using a sign, the significant female odds ratio excess for securing a first, co- and last authorship. For example, the FAOR-triplet (+, –, =) indicates that women have significantly higher odds for first-authorships, lower odds for co-authorships and equal odds for last authorships. To summarize, the quantitative representation of female authorships is measured by the FAP, while the three FAORs quantify the relative distribution of female authorships among all the different authorships [23, 24]. The FAP/FAOR-classification is only conducted for subjects (e.g. countries) with a minimum of 750 male or female authorships, so that adequate statistical precision in terms of small confidence intervals can be achieved. For journal classification see Supplementary Table 1.

### Prestige index

The *Prestige Index* measures the female odds excess, compared to that of men, for securing prestigious authorships [24]. The *Prestige Index* is defined as the prestige-weighted average of the FAOR excess ε_t_ that is calculated over all authorship types, t (i.e. for first, co- and last authorships), ε_t_ = w_t_ (FAOR_t_ – 1), if FAOR_t_^3^ 1, otherwise ε_t_ = w_t_ (1–1/FAOR_t_) with the weighting factor w_t_ [23]. As first and last authorships have a higher effect on reputation than co-authorships, they were graded positively (w_first_ = w_last_ = 1), whereas co-authorships were graded negatively (w_co_ = –1) Due to this weighting scheme, lower odds for a middle authorship increase the *Prestige Index*, whereas lower odds for a first or last authorship decrease the *Prestige Index*. A *Prestige Index* of 0 indicates a gender-neutral distribution of prestigious authorships, whereas a value above 0 indicates an excess of prestigious authorships, a value below 0 a lack of prestigious authorships held by women [23, 24]. An additional test was used to exclude an alphabetic ordering of the author list (Supplementary Figure 5) [24].

### Analysis of data

Annual growth was measured using average annual growth rates (AAGR) that are defined as the geometric mean of *n* annual grow rates *p*, $\text{AAGR}={\left[\prod_{t=1}^{n}\left(1+p_{t}\right)\right]}^{1/n}-1$. The AAGRs of the article count and the FAPs were used to create a linear forecast of the temporal development of FAP, FAOR and the *Prestige Index* for the next 6 years. The Global Gender Report's linear association between FAP, *Prestige Index* and *Score* [42] was evaluated using the Pearson correlation. The null hypothesis whether the abnormally distributed citation rates of the different article groups (Supplementary Figure 4) are drawn from the same distribution, was tested using both a Kruskal-Wallis and a follow-up multiple comparison test [23, 24].

## SUPPLEMENTARY MATERIALS FIGURES AND TABLE


