# Supplementary material for: Gendermetrics of cancer research: results from a global analysis on prostate cancer
Source: Oncotarget. 2018 Apr 13;9(28):19640–9. doi: 10.18632/oncotarget.24716 (PMC5929414; doi:10.18632/oncotarget.24716)
Supplement: Supplementary file 1 [file oncotarget-09-19640-s001.pdf]

Gendermetrics of cancer research: results from a global analysis on prostate cancer

SUPPLEMENTARY MATERIALS

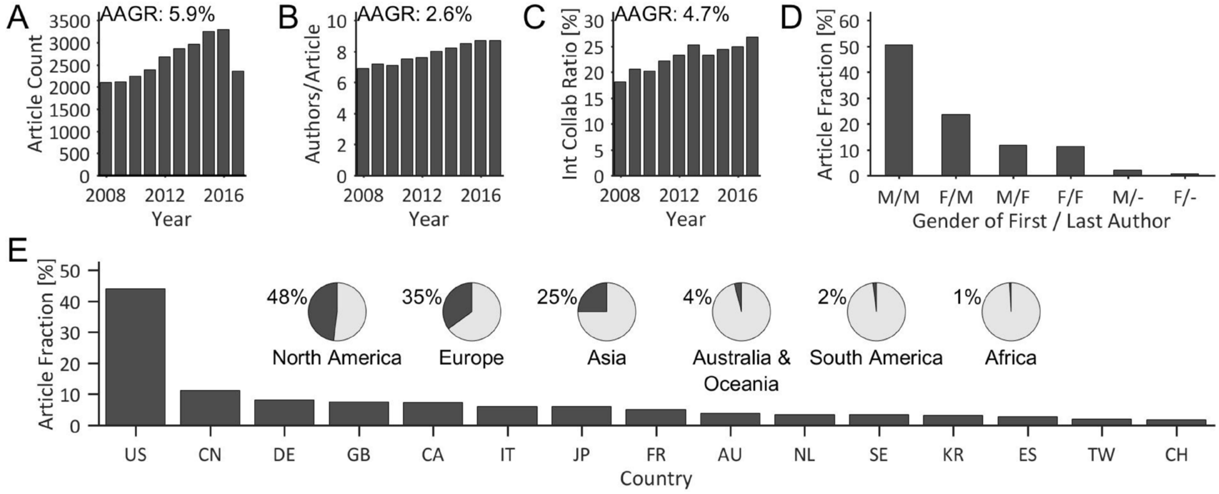

Supplementary Figure 1: Bibliometric Overview. The study period covers January 1, 2008 to September 12, 2017, yielding 26,234 articles. (**A**) The article count exhibits an average annual growth rate (AAGR) of 5.9%. (**B**) The number of authors per article increases from 6.9 authors/article in 2008 to 8.7 authors/article in 2017 with an AAGR of 2.6%. (**C**) The ratio of international collaboration articles increases from 18.1% in 2008 to 24.9% in 2017, which results in an AAGR of 4.7%. (**D**) The grouping of articles by the gender of their first and last author reveals a quantitative dominance of combined male first and last authorships (M/-, F/-: single authorships). (**E**) The fraction of articles by country (bar plot) and continent (pie chart) documents that the United States (44.0%), China (11.3%) and on the level of continents, North America (48%), Europe (35%) and Asia (25%) are the most productive countries and continents, respectively. Please note that the sum of ratios is greater than one in both group classes due to international collaborations. AU = Australia, CA = Canada, CH = Switzerland, CN = China, DE = Germany, ES = Spain, FR = France, GB = United Kingdom, IT = Italy, JP = Japan, KR = South Korea, NL = The Netherlands, SE = Sweden, TW = Taiwan, US = United States.

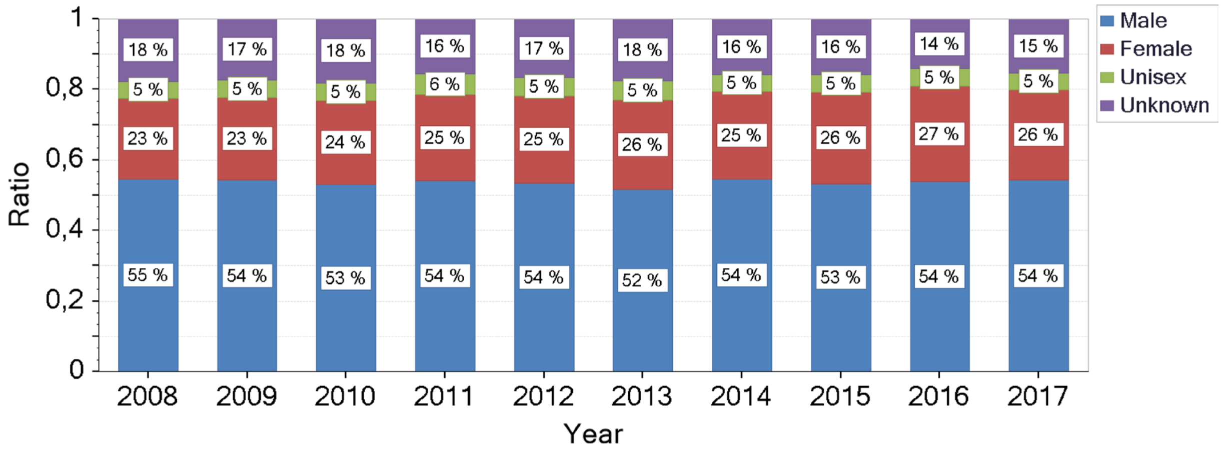

Supplementary Figure 2: Gender detection output by time. Relatively little inter-annual variability characterizes the ratios of detected male, female, unisex and undefined authorships.

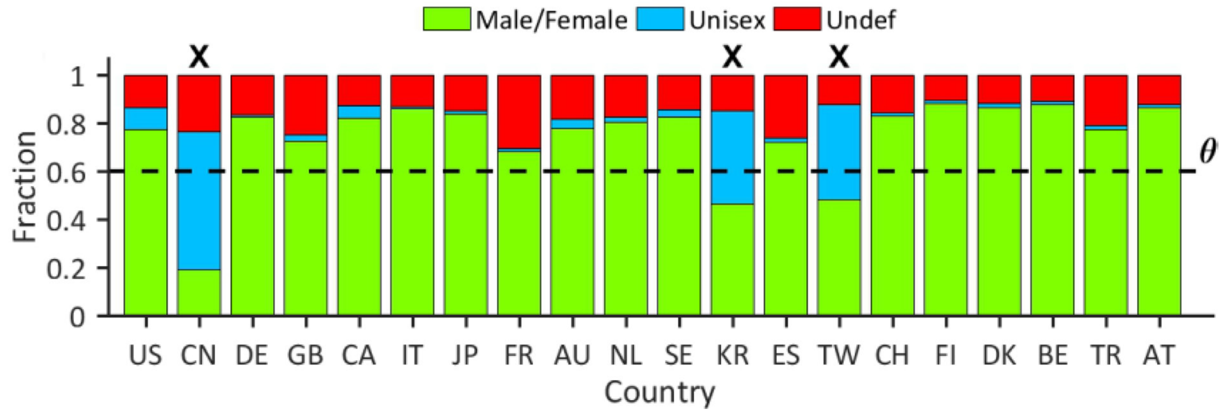

Supplementary Figure 3: Quality of algorithmic gender detection by country. The result of the algorithmic gender detection by country documents a relative high frequency of male/female authors for most of the top 20 countries, with the exception of the Asian countries China, South Korea and Taiwan. The latter countries are characterized by a high frequency of unisex names and were excluded (X) from analysis due to the threshold criterion of $\theta = 60\%$ male or female authorships (dashed line). AT = Austria, AU = Australia, BE = Belgium, CA = Canada, CH = Switzerland, CN = China, DE = Germany, ES = Spain, FI = Finland, FR = France, GB = United Kingdom, DK = Denmark, IT = Italy, JP = Japan, KR = South Korea, NL = The Netherlands, SE = Sweden, TR = Turkey, TW = Taiwan, US = United States.

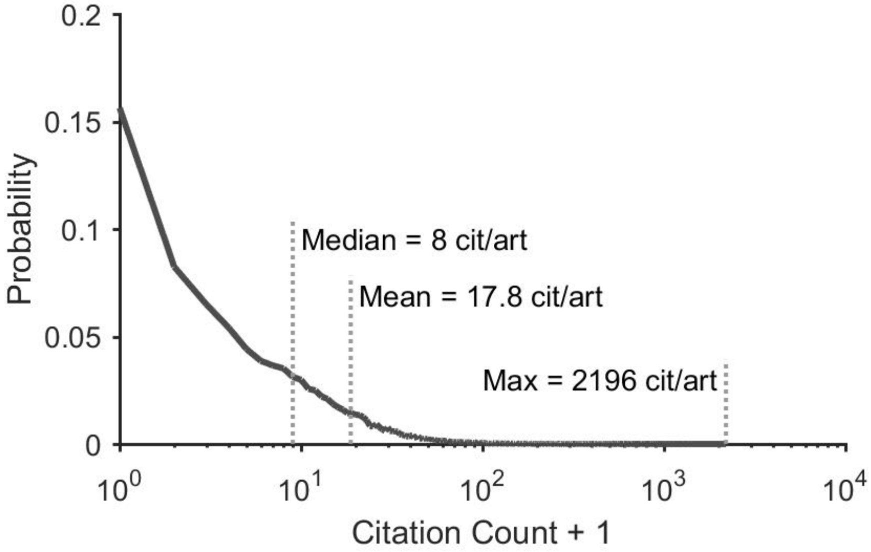

Supplementary Figure 4: Probability density function of the citation rate. The semi-logarithmic plot of the citation count per article (=citation rate) shows an exponential-like decreasing probability density function with a mean citation rate of 17.8 citations/article.

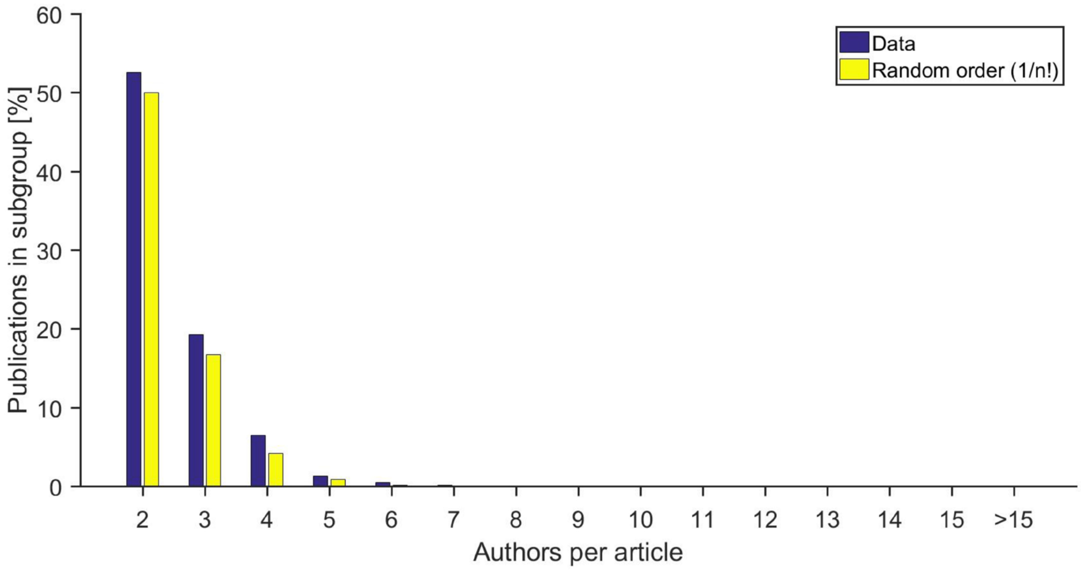

Supplementary Figure 5: Test for alphabetical ordering of the author list. The proportion of publications with an alphabetic ordered author list is depicted with respect to the authors per article (black). The values correspond very closely to those obtained for randomly ordered author lists (gray).

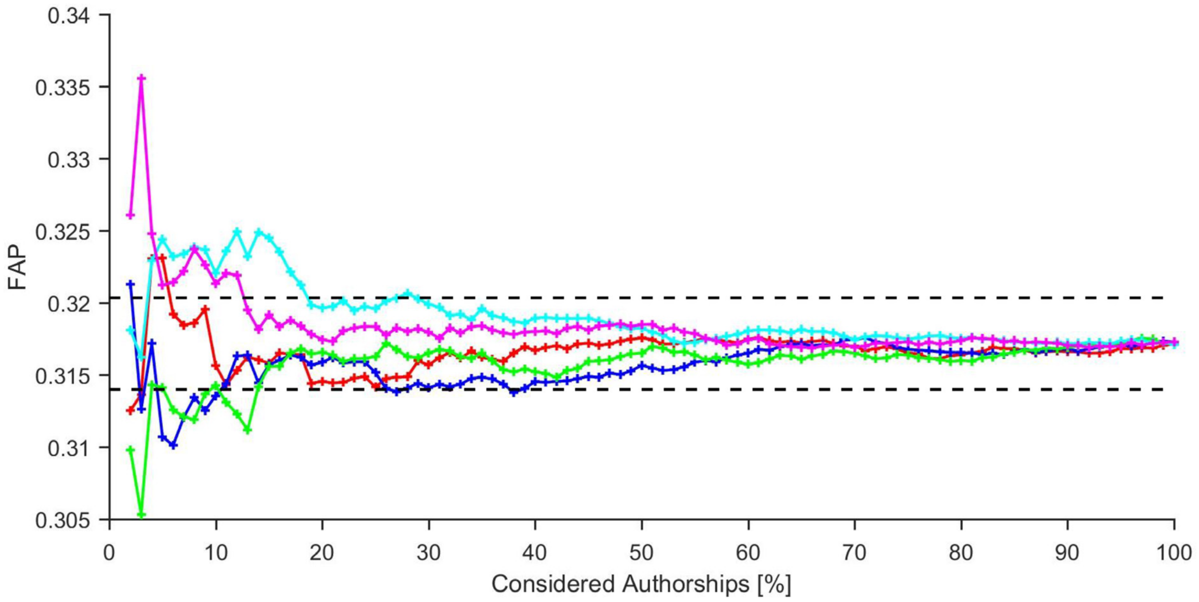

Supplementary Figure 6: Convergence of gender determination. The figure shows the FAP as a function of the considered authorships in 5 consecutive runs (authorships were shuffled before each run). The dashed lines represent the 1% interval around the effective FAP. Evidently, the algorithm converges very fast; it is sufficient to consider about 20% of all *male and female* authorships to assess the FAP with an error deviation of less than 1%.

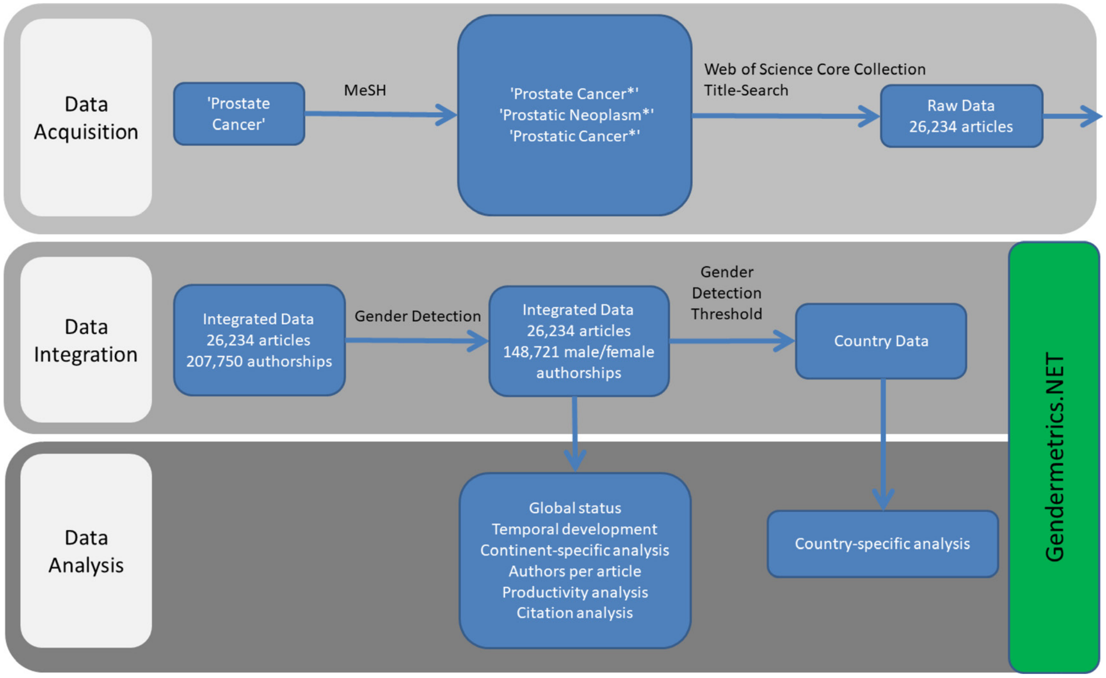

Supplementary Figure 7: Flow diagram of procedure steps. The synonyms for 'Prostate Cancer' were determined by the MeSH library (Medical Subject Headings) of the National Library of Medicine. English-language research articles were acquired from the Web of Science Core Collection by performing a title search. Data Integration and Data Analysis was conducted using Gendermetrics.NET [1]. Unisex and undefined authors and their authorships were ignored in further analysis. In total, N = 148,721 male and female authorships form the database for the analysis. In order to ensure the validity of the country-specific analysis a gender detection threshold criterion for the inclusion of a country was applied. Specifically, countries with a detection fraction below 60.0% male and female authorships were excluded from this subanalysis.

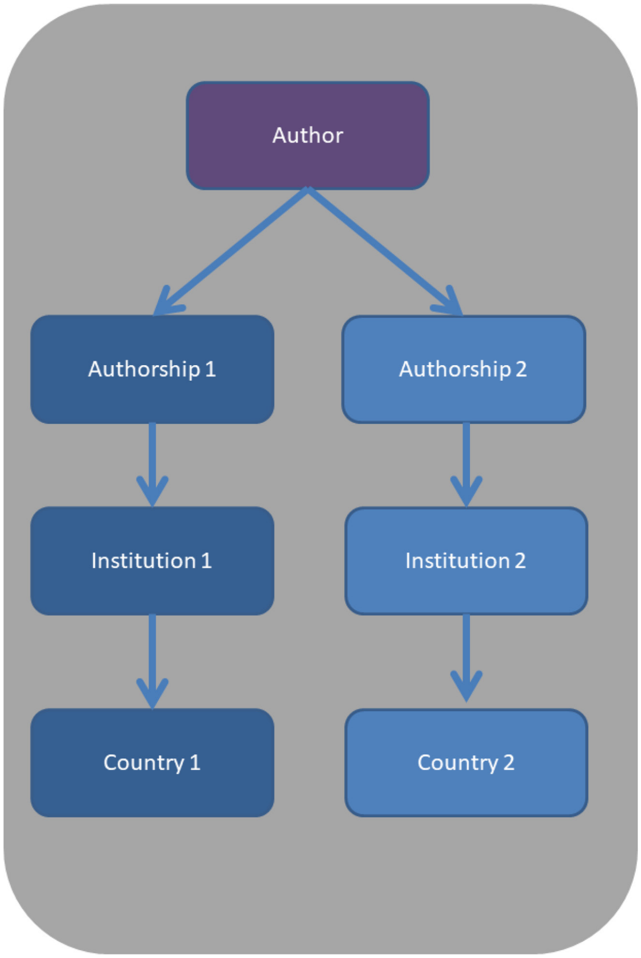

Supplementary Figure 8: The concept of authors and authorships. The research output of a country was determined on the basis of the associated institutions and their authorships. A single author is thus able to contribute to the research output of different countries.

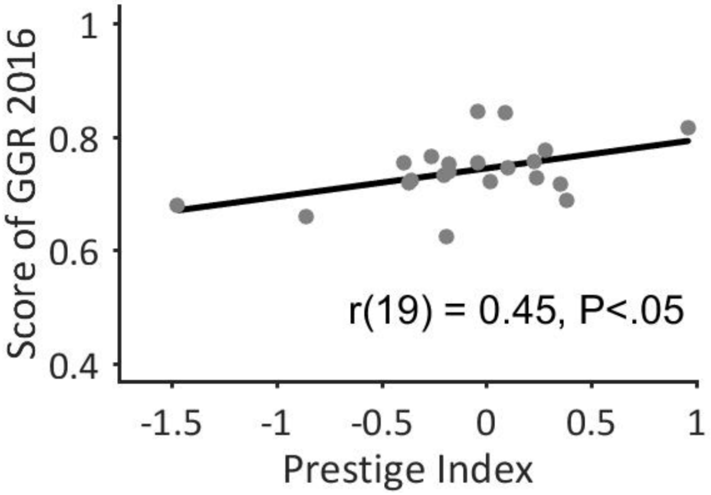

Supplementary Figure 9: *Prestige Index* vs. Score of Global Gender Report 2016. We reveal a moderate linear correlation between the Prestige Index of a country and its Score defined by the Global Gender Report 2016. Major regional differences stem primarily from the socio-cultural surroundings of a country and are not the outcome of discipline-specific characteristics

Supplementary Table 1: Classification of most productive journals that were descendingly ordered by the *Prestige Index*

Journal Name	*Prestige Index*	FAP	FAOR Triplet	No. Articles	No. Authorships
Human Molecular Genetics	0.72	38.5%	(=, =, =)	27	875
Cancer Causes & Control	0.64	45.9%	(+, -, =)	147	987
International Journal of Oncology	0.32	35.4%	(+, =, =)	190	942
Cancer Epidem Biomarkers & Prevention	0.26	45.2%	(+, =, =)	232	2,351
International Journal of Cancer	0.24	37.6%	(+, =, -)	299	2,407
Oncotarget	0.10	37.6%	(+, =, -)	554	3,729
Bmc Cancer	0.08	37.9%	(+, =, =)	246	1,727
Plos One	0.07	38.9%	(+, =, -)	726	4,622
Journal Of Nuclear Medicine	0.05	24.4%	(+, =, -)	115	933
Europ Journal of Nuc Med And Mol Imag	-0.08	25.1%	(=, =, =)	112	928
Molecular Cancer Therapeutics	-0.09	32.8%	(+, =, -)	131	847
European Journal of Cancer	-0.09	36.3%	(=, =, =)	126	801
Prostate	-0.11	34.9%	(+, =, -)	1,155	7,287
Lancet Oncology	-0.13	26.6%	(=, =, =)	73	1,069
Cancer Research	-0.19	34.1%	(+, =, -)	379	3,041
Urologia Internationalis	-0.25	15.8%	(=, =, =)	160	933
Carcinogenesis	-0.27	39.1%	(=, =, =)	119	823
Radiation Oncology	-0.31	28.0%	(=, =, -)	147	1,141
Int Journal of Radiation Oncol Biol Physics	-0.32	26.3%	(=, +, -)	504	3,831
Clinical Cancer Research	-0.34	30.9%	(=, +, -)	349	3,088
Radiotherapy and Oncology	-0.38	30.9%	(=, +, -)	224	1,807
Bju International	-0.45	24.0%	(=, +, -)	761	5,489
Anticancer Research	-0.45	22.9%	(=, =, -)	256	1,583
Journal of Clin Oncol	-0.46	29.0%	(=, +, -)	204	2,250
European Urology	-0.5	23.5%	(=, +, -)	501	5,059
Brachytherapy	-0.57	22.6%	(=, +, -)	132	858
Urologic Oncology-Sem And Orig Investig	-0.63	26.7%	(=, +, -)	378	2,304
World Journal of Urology	-0.64	22.2%	(=, +, -)	236	1,418
Int Journal of Urology	-0.81	12.8%	(=, +, -)	152	1,027
Urology	-0.88	23.6%	(=, +, -)	446	2,851
Journal Of Urology	-0.89	23.5%	(=, +, -)	619	4,221
Cancer	-0.89	27.8%	(=, +, -)	280	1,999
Nature Genetics	-0.91	36.3%	(=, =, =)	29	1,230
Clinical Genitourinary Cancer	-1.31	31.3%	(-, +, -)	162	1,131
PNAS	-1.91	32.6%	(=, +, -)	82	818

SUPPLEMENTARY REFERENCE

1. Bendels MHK, Brüggmann D, Schöffel N, Groneberg DA. Gendermetrics. NET: a novel software for analyzing the gender representation in scientific authoring. J Occup Med Toxicol. 2016; 11:43.
